# Assessment of Treatment Effectiveness in Acute and Chronic Anal Fissures

**DOI:** 10.3390/medicina62030490

**Published:** 2026-03-05

**Authors:** Onur İlkay Dinçer, Duygu Felek, Erol Cakmak, Vugar Ali Turksoy

**Affiliations:** 1 Department of General Surgery, Istinye University Medical Park Antalya Hospital, 07000 Antalya, Türkiye; onurilkay.dincer@medicalpark.com.tr; 2 Department of Internal Medicine, Faculty of Medicine, Yozgat Bozok University, 66100 Yozgat, Türkiye; duygu.felek@yobu.edu.tr; 3 Department of Gastroentrology, Faculty of Health Sciences, Fenerbahce University, 34000 Istanbul, Türkiye; erol.cakmak@fbu.edu.tr; 4 Department of Public Health, Faculty of Medicine, Yozgat Bozok University, 66100 Yozgat, Türkiye

**Keywords:** anal fissure, antibiotherapy, metronidazole

## Abstract

*Background and Objectives:* Anal fissures are a common condition in the general population, for which there are multiple treatment options. It is essential to select the most appropriate treatment for the right patient. This study aimed to observe and evaluate the effect of topical antibiotherapy, which is widely used in the management of wounds and chronic infections, on the healing of acute and chronic anal fissures. *Materials and Methods:* Hospital records of 625 individuals diagnosed with an anal fissure were reviewed. Previous treatments, including 0.4% glyceryl trinitrate and 5% lidocaine, were recorded. A total of 118 patients were included: 49 patients who received additional topical metronidazole due to inflammation, induration and minimal purulent discharge, in the absence of an abscess; and 69 uncomplicated patients who received only standard treatment, as per the exclusion criteria. *Results:* The mean age of the participants was 41.06 ± 10.70 years. No significant differences were found between the groups regarding age or sex (*p* = 0.616 and *p* = 0.665, respectively). However, prior treatment history and mucosal healing differed significantly between the two groups (*p* = 0.001 and *p* = 0.024, respectively). There were no significant differences in follow-up intervals, additional treatment requirements or improvement in VAS scores (*p* = 0.546, 0.904 and 0.154, respectively). *Conclusions:* Topical metronidazole may be associated with improved mucosal healing in selected patients with acute anal fissures presenting with clinical features such as local inflammation, minimal discharge or incision-related changes. However, the observed benefit does not appear to be uniform across all patients, and, in the absence of microbiological data, the extent of microbial involvement remains uncertain. Accordingly, topical metronidazole may be considered for carefully selected cases of acute anal fissure based on clinical judgement, while avoiding routine or indiscriminate antibiotic use.

## 1. Introduction

An anal fissure is a common benign anorectal disorder characterised by a linear tear in the anoderm that extends distally from the dentate line towards the anocutaneous junction. It typically presents with sharp pain during and after defecation, and bright red rectal bleeding and discomfort, which can significantly impair quality of life [1]. Epidemiological data suggest an annual incidence of approximately 1.1 per 1000 individuals, with a lifetime risk estimated at 7–8%, indicating the considerable burden of this condition on the general population [2]. The precise aetiology of anal fissures is multifactorial and incompletely understood, but several mechanisms have been implicated, including local trauma from hard stools, sphincter hypertonicity, mucosal ischaemia and chronic inflammatory processes [1,2,3]. One of the underlying aetiological mechanisms is hypertonic spasm of the internal anal sphincter and ischaemia, particularly affecting the parasympathetic cholinergic nerve pathways that regulate sphincter tone. This contributes to muscle relaxation via acetylcholine acting on muscarinic receptors [4]. Caffeine has a stimulatory effect because it blocks adenosine receptors, and some in vitro studies have reported that it increases acetylcholine release [5]. Moderate coffee consumption (less than four cups per day) may enrich the gut microbiota, increase the proportion of beneficial bacteria and reduce the risk of constipation by regulating stool transit. The current literature suggests that moderate (4 cup and less) coffee consumption has beneficial effects on oral and gut microbiota and motility function [6]. Manometric studies have demonstrated that caffeine intake increases anal sphincter pressure and alters rectal sensitivity. This suggests that caffeine has indirect effects on anorectal smooth muscle and neural control. However, by stimulating intestinal motility, high caffeine intake may increase sensitivity and irritation. Therefore, limiting coffee consumption during the active phase of anal fissure is recommended [7]. The effect of diet on etiology should also be evaluated, particularly considering factors that can affect bowel motility and the enteric system, such as water, milk, and coffee. In this context, it is thought that insufficient consumption of these liquids may reduce motility stimuli, which in turn may lead to hardening of stool consistency and increased trauma during defecation, creating a favorable environment for the development of fissures [8]. These findings emphasise that medical treatment and individual dietary habits should both be considered when managing anal fissures.

Anal fissures often begin with an acute tear, which may evolve into a chronic lesion if mucosal healing is not achieved within four to six weeks. Around 40% of acute fissures progress to chronic fissures, highlighting the importance of timely and effective management [3]. Chronic anal fissures are typically deeper, with exposed internal anal sphincter fibres and indurated fissure edges. They are often accompanied by hypertrophic anal papillae proximally and sentinel skin tags or haemorrhoids distally, reflecting long-standing irritation and failed wound-healing responses [9,10]. Current clinical guidelines, including those from the American Society of Colon and Rectal Surgeons (ASCRS), recommend conservative therapy as the primary treatment for acute fissures. Such measures include warm sitz baths, dietary fibre optimisation, stool softeners and topical analgesics. Especially warm water sitz baths, implemented among conservative approaches historically, are also used for pain and spasm management in anal fissure management [11]. Using dilators alongside antibiotics, or immediately afterwards, for an extended period of time and with adequate lubrication using ointments containing calcium channel blockers can alleviate symptoms and speed up the healing process when administered twice daily. Pharmacological therapies, such as topical nitroglycerin, calcium channel blockers and, in selected cases, botulinum toxin, aim to reduce internal anal sphincter pressure and improve mucosal perfusion [12,13,14]. When medical and conservative treatments fail, surgical intervention, particularly lateral internal sphincterotomy (LIS), remains the gold standard due to its high success rates. However, LIS is associated with potential complications, including gas or faecal incontinence, which has been reported in 3.4–4.4% of cases. Some studies have noted mild long-term continence disturbances in up to 47% of patients [15,16]. Postoperative pain, local infection and persistent or recurrent fissure are additional concerns [17].

In light of these limitations, there has been an increasing shift towards optimising non-surgical treatment strategies and identifying patient subgroups who may benefit from additional therapies. One area of growing interest is the suspected role of chronic low-grade infection in the pathogenesis and impaired healing of anal fissures. Chronic wounds, including fissures that fail to heal, are known to share several key features: persistent inflammation, impaired angiogenesis, bacterial overgrowth and dysregulated epithelial repair [15,18,19]. These mechanisms may explain why some fissures do not respond to standard vasodilatory or sphincter-relaxing treatments. Topical and oral antibiotics have therefore been proposed as potential adjuncts in selected patients. Several studies have suggested that antimicrobial therapy may reduce bacterial load, alleviate local inflammation and promote faster mucosal recovery, particularly in fissures exhibiting features of secondary infection, such as discharge, induration or inflammatory oedema [17]. Metronidazole, in particular, has attracted attention due to its anaerobic antibacterial spectrum and its anti-inflammatory properties. Evidence suggests that topical metronidazole can alleviate pain and promote faster healing in acute fissures, making it a promising option in carefully selected clinical scenarios [20]. Despite these findings, the routine use of topical antibiotics is not currently included in standard fissure management guidelines, primarily due to a lack of high-quality evidence and concerns about unnecessary antibiotic exposure. Therefore, identifying which patients may benefit from additional antimicrobial therapy remains an important yet unresolved clinical question. Against this backdrop, our study sought to evaluate the treatment modalities employed in the real world for managing anal fissures and to retrospectively assess healing outcomes in patients prescribed topical antibiotherapy due to clinical signs of inflammation.

## 2. Materials and Methods

### 2.1. Ethics Approval and Study Design

After receiving local ethics approval (2017-KAEK-189_2022.12.29_02), the study was initiated. Hospital records of 625 patients diagnosed with an anal fissure and who presented at the General Surgery Clinic of Yozgat Sorgun State Hospital between 2015 and 2022 were reviewed. Previous treatments, including 0.4% glyceryl trinitrate and 5% lidocaine, were recorded. A total of 51 patients were prescribed additional topical metronidazole due to inflammation, induration and minimal purulent discharge without abscess. Of these patients, one was excluded due to malignancy detected during colonoscopy and another due to lack of follow-up, leaving 49 patients for inclusion. Of the remaining 574 patients, only 69 met the inclusion criteria and had adequate follow-up. In total, 118 patients were included. Patients were classified as acute or chronic based on hospital records and physical examination notes. Those receiving standard therapy and those receiving additional topical metronidazole were further subdivided (no wound culture examination was found in the database). Patients who continue treatment after the standard treatment period have been identified as requiring additional treatment [19,20].

### 2.2. Acute—Chronic Anal Fissure

An acute anal fissure is defined as a linear tear in the distal anoderm. A chronic anal fissure is characterised by indurated edges, exposure of internal anal sphincter fibres, a hypertrophic papilla and/or a sentinel pile [21,22]. Complete mucosal healing of an anal fissure is defined as the fissure’s complete physical closure, the restoration of superficial mucosal integrity, and the absence of granulation tissue and exudate at the fissure’s base [23].

For this study, patients with chronic anal fissures, as recorded in previous examinations, who exhibited at least one of the following findings—indurated edges, exposure of internal anal sphincter fibres, hypertrophic papillae or a sentinel pile, or who had a history of prior treatment for anal fissures—were included in the chronic anal fissure group. Patients diagnosed with acute anal fissure and whose symptom duration was less than six weeks were included in the acute anal fissure group.

### 2.3. Visual Analog Scale (VAS)

The visual analogue scale (VAS) is a scoring system in which patients report their pain on a scale from 0 (no pain) to 10 (worst possible pain). Pain was assessed using a VAS ranging from 0 to 10.

### 2.4. Exclusion Criteria

Patients with a diagnosis or examination note indicating perianal fistula, abscess or haemorrhoidal disease, or who had undergone perianal surgery, were excluded from the study. Patients with a history of inflammatory bowel disease, malignancy, or chemotherapy or radiotherapy were also excluded. Individuals with inflammatory bowel disease or malignancy, as detected by rectoscopy or colonoscopy, were also excluded. Patients without post-treatment follow-up records were also excluded.

### 2.5. Statistical Analysis

All statistical analyses were performed using SPSS version 20.0 (IBM, New York, NY, USA). Prior to hypothesis testing, all variables were examined for data quality, missing values and distributional characteristics. Continuous variables (e.g., age, BMI, VAS scores and follow-up duration) were tested for normality using the Shapiro–Wilk test and supported by Q–Q plots, histograms and skewness/kurtosis values. As most variables did not meet the assumptions of parametric tests, non-parametric statistical methods were used instead. Continuous variables are presented as mean ± standard deviation (SD) or median (interquartile range, IQR) as appropriate. Categorical variables (sex, smoking status, mucosal healing, need for additional treatment, prior treatment history and acute/chronic classification) are reported as frequencies and percentages. As normality assumptions were not met, comparisons were performed using the Mann–Whitney U test. Differences in proportions were evaluated using the Pearson chi-squared test or Fisher’s exact test when the expected cell count was less than five.

Separate analyses were conducted for acute and chronic cases to evaluate differences in the effect of treatment. Within each subgroup, comparisons between antibiotic-treated and non-treated patients were performed using Pearson’s chi-squared test or Fisher’s exact test for categorical variables, and the Mann–Whitney U test for continuous variables. Two-by-two pairwise analysis (post hoc comparisons). When the overall chi-square test was significant, Bonferroni-adjusted pairwise comparisons were applied to control for type I error. For variables measured at two time points (e.g., VAS improvement, initial vs. follow-up evaluation): The McNemar test was used for paired categorical variables, and the Wilcoxon signed-rank test was used for paired continuous variables (e.g., VAS change score). Due to non-normal data structure, relationships between continuous variables (e.g., BMI, age, follow-up duration and VAS scores) were assessed using Spearman’s rank correlation coefficient (ρ). Although this was a retrospective study, post hoc power calculations were performed for the main outcomes (mucosal healing and VAS improvement) to determine whether the sample sizes in the acute and chronic subgroups were large enough to detect clinically significant differences with 80% power. Significance was set at *p* < 0.05.

## 3. Results

Examination of the hospital record system data for 118 patients who met the inclusion and exclusion criteria and had complete follow-up notes revealed that they could be divided into acute and chronic anal fissure subgroups according to their diagnosis codes, based on the use of topical antibiotic therapy due to inflammation, induration and minimal purulent discharge. Examining all patients revealed that there were more males than females. The minimum and maximum ages of patients included in the study were 18 and 75 years, respectively, with an average age of 41.95 ± 10.97 years. No significant differences were observed in terms of age and gender between groups when the age and gender data were evaluated (*p*-values were 0.616 and 0.665, respectively). Using the hospital data system, no statistically significant difference was found in the time between the initial examination and the start of treatment (*p* = 0.354). No statistical difference was observed between the groups in terms of fissure localisation (*p* = 0.911). Similarly, no statistical difference was observed between the groups in terms of the medical treatments they received, such as glyceryl trinitrate, diltiazem hydrochloride, analgesic treatments, and conservative approaches (*p* = 0.283). No statistically significant difference was found when the groups were compared according to the fissure line (*p* = 0.911). No statistically significant difference was observed between patients in the system when they were compared according to the types of treatment they had previously received (*p* = 0.367). When the smoking habits of patients were evaluated across groups, the highest rate (54.5%) was found among individuals with chronic fissures. This difference was statistically significant (*p* = 0.021). However, when alcohol consumption rates were evaluated based on the available data, no statistically significant difference was observed (*p* = 0.456). No significant difference was observed when the groups were compared in terms of body mass index (*p* = 0.092) (Table 1).

**Figure 1:**
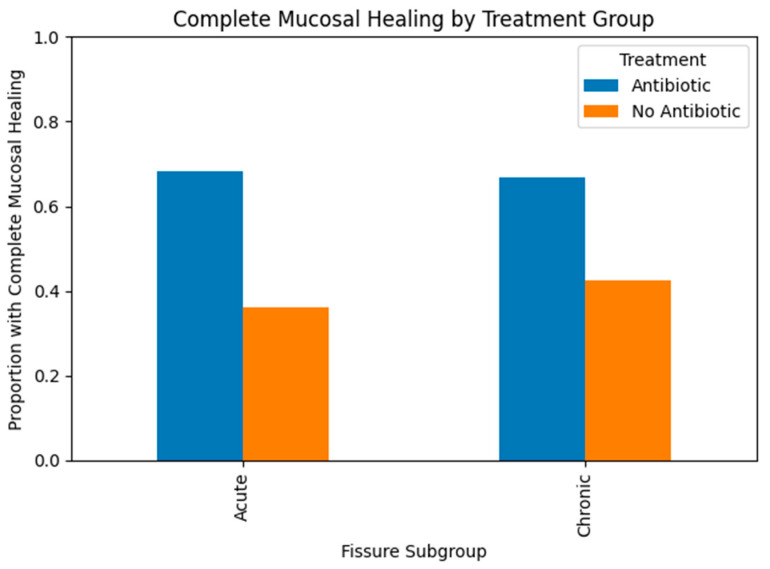
Mucosal healing rates in acute and chronic anal fissure subgroups according to treatment modality.

A significant difference was observed when evaluating whether patients in the groups had previously received treatment and their mucosal healing status (*p* = 0.001; 0.024, respectively). No statistically significant differences were observed between the groups in terms of the time taken for patients to attend follow-up appointments, the need for additional treatment, or improvement rates in VAS scores (*p* = 0.546; 0.904; 0.154, respectively). When the groups were compared in pairs within meaningful parameters, a higher rate of mucosal healing was observed in the group that received antibiotic treatment for an acute anal fissure than in the untreated group (*p* = 0.017). Although the difference was higher in the chronic group, it was not statistically significant (*p* = 0.074) (Figure 1).

**Figure 2:**
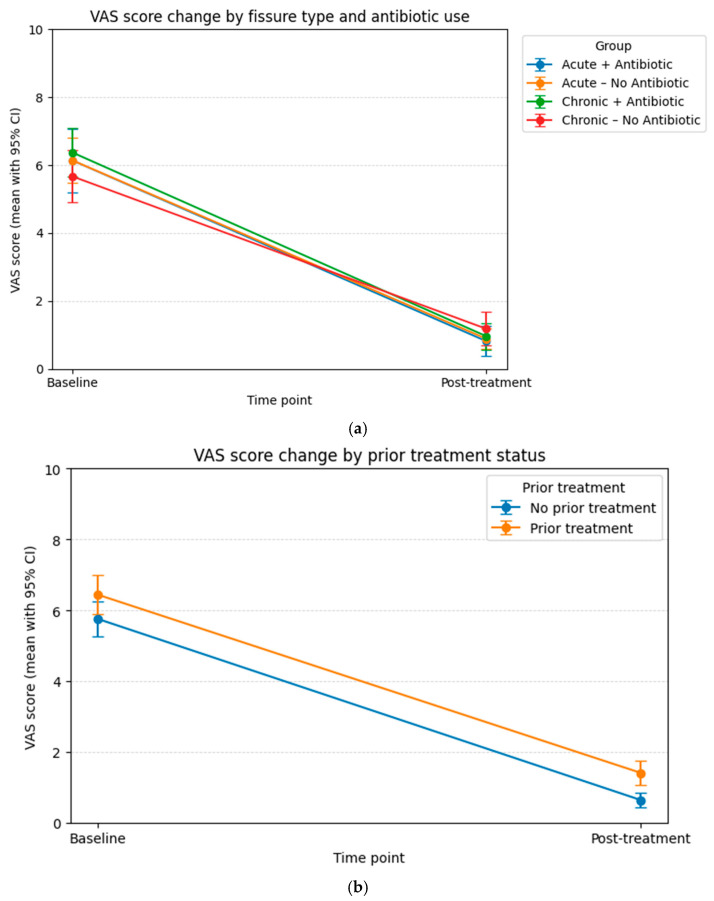
VAS score change by antibiotic using status (**a**) and VAS score change by prior treatment status (**b**).

Examining the relationship between VAS scores, treatment options and past treatment use revealed a decrease in VAS scores in each group. However, no statistically significant difference was found between antibiotic use, prior treatment, and the rate of improvement in VAS scores (Figure 2a,b). While no significant differences in VAS improvement were observed between groups, these findings do not provide definitive evidence regarding the effect of antibiotics on pain. The results may have been influenced by confounding factors, the limited sample size and concurrent treatments that affect pain perception.

Although there is a positive correlation between age and the group variable, this relationship is not statistically significant (B = 0.012, β = 0.119, *p* = 0.253). A positive effect was observed for the BMI (BMI^2^) variable, with a result close to the threshold for statistical significance (B = 0.039, β = 0.181, *p* = 0.068). These findings suggest that the group variable tends to increase with BMI but indicate that a larger sample size is required to establish a definitive relationship. The post-treatment VAS score has a positive, albeit non-significant, effect on the group variable (B = 0.128, β = 0.134, *p* = 0.179). Gender does not significantly predict the group variable in the model (B = −0.031, β = −0.014, *p* = 0.885). Smoking shows a negative relationship with the group variable and approaches statistical significance (B = −0.382, β = −0.173, *p* = 0.106). This suggests that smoking may have a suppressive effect on the group variable. Alcohol use has no significant effect on the group variable (B = −0.034, β = −0.012, *p* = 0.903). VAS improvement shows a negative but statistically insignificant relationship with the group variable (B = −0.029, β = −0.048, *p* = 0.626). Multiple linear regression analysis was performed to evaluate the effects of the following variables on the group as the dependent variable: age, BMI, post-treatment VAS score, gender, smoking, alcohol consumption and VAS improvement. When all the independent variables included in the model were evaluated together, no statistically significant predictor variables were identified (*p* > 0.05). However, some variables were found to be borderline significant.

The treatment effects were quantified using risk ratios with 95% confidence intervals. These were used to evaluate the precision of the estimates and to determine whether the observed differences between acute and chronic fissures were consistent with clinically significant effects. Examining the effect sizes revealed that topical antibiotic therapy had a stronger impact on acute fissures (RR = 1.89, 95% CI 1.10–3.25; +32.1%) than on chronic fissures (RR = 1.57, 95% CI 0.94–2.62; +24.3%). While the direction of the effect remained consistent across both subgroups, statistical significance was only achieved in cases of acute fissures. This suggests that reduced power and chronic wound characteristics may have caused the lack of significance in chronic cases, rather than an absence of therapeutic effect.

## 4. Discussion

Our study found that a large group of patients undergoing treatment for anal fissures were excluded because they did not regularly attend follow-up appointments and their notes were insufficient. This situation demonstrates a high level of non-compliance with treatment, indicating that this is one of the main causes of chronicity. Furthermore, given that some patients did not start treatment or return for follow-ups, it is thought that many could have been treated through lifestyle changes and dietary adjustments. It was observed that the group experiencing inflammation, induration and minimal pruritic perianal discharge attended follow-up appointments more regularly. This suggests that this group of patients may have specifically been warned by their physician about potential complications. Chiu et al.’s study described anal fissure treatment modalities, listing options such as topical nitrates, calcium channel blockers, botulinum toxin, and surgery. However, they emphasised the importance of patient-specific treatment and mentioned special circumstances [24]. Treatment that is not tailored to the individual may also cause chronicity. In his study summarising the management of anal fissures, Higuero primarily addressed lifestyle changes and treatment duration, followed by surgical options and their associated risks in cases where medical treatment is unsuccessful. Given the wide range of treatment options for this condition, it makes sense to opt for the least invasive method. It is also necessary to determine the most appropriate treatment for each patient to prevent chronicity and the development of complications [25]. The higher incidence of anal fissures in men compared to women in patient records is consistent with the literature, and no gender-based differences have been observed in acute or chronic fissures. Current studies focus more on which gender is affected more severely by complications. Although complicated cases are more frequently reported in males, studies showing no gender difference can also be found [26,27,28]. The average age at which anal fissures occur is 41.95 years, which is consistent with the literature. The frequent occurrence of anal fissures in the middle-aged group is indicative of age-related factors. The higher average age of patients with chronic anal fissures compared to those with acute anal fissures is thought to be due to an increased rate of chronic fissures with age in later periods, caused by acute anal fissures that are not adequately treated. In a study by Lida et al., the average age for chronic anal fissure was found to be 50.16 years [29]. With advancing age, the reduction in anal sphincter muscle tone leads to relative sphincter relaxation, which may hinder the establishment of the mechanical conditions required for fissure formation, thereby decreasing the likelihood of anal fissure development [13]. Prolonged inflammation and recurrent infections, which are also influenced by advancing age, can lead to chronic perianal fistula disease. It is known that anal fissures in which complete mucosal healing is not achieved and which sometimes develop abscess-infective complications can lead to perianal fistulas and may require a different surgical approach [30]. Over time, this disease can result in complications such as anal canal narrowing (anorectal stenosis) due to the development of fibrosis and scar tissue in the anorectal region [31]. Although anal fissures are less frequently observed during childhood, similar to advanced age, chronic inadequate fluid intake has been reported to increase the risk in later stages of life [32]. Furthermore, the volume of fluid intake beginning in the breastfeeding period has been identified as a potential risk determinant; breastfeeding appears to exert a protective effect, whereas cow’s milk consumption has been associated with an elevated risk of anal fissure development [33]. Collectively, existing evidence highlights the contributory roles of age-related physiological changes and nutritional factors in the pathogenesis of anal fissures. From infancy to old age, the primary goal should be to eliminate risk factors associated with anal fissures at every stage of life to ensure healing before chronicity develops. This is because chronic fistulising perianal disease and inflammation-related fibrosis can lead to narrowing of the anal canal and impaired function, making treatment more complex and negatively affecting quality of life [34]. In elderly patients, the cumulative impact of persistent or recurrent anal fissures can extend beyond symptomatic discomfort to induce structural changes within the anal canal. Repeated cycles of inflammation and tissue repair promote excessive collagen deposition and fibroblast activation in the anoderm and internal sphincter complex, gradually replacing normally elastic tissue with dense fibrotic scar. Age-related reductions in tissue elasticity and impaired regenerative capacity further amplify this process, increasing the risk of a fixed anatomical stenosis. Over time, such fibrotic remodeling can compromise anal canal compliance, hinder normal function, and make subsequent management more challenging, often necessitating more complex interventions when conservative measures prove insufficient [35,36].

The development of acute and chronic anal fissures was evaluated in relation to body mass index, environmental factors such as smoking and alcohol consumption, and dietary habits; body mass index was not a distinguishing factor. This finding lends weight to the idea that obesity does not contribute to the chronicity of fissures. However, a comparison between a healthy control group and a group with similar characteristics but without anal fissures is needed to determine whether obesity plays a role in the development of anal fissures. The effect of caudal epidural injection on the healing process in chronic anal fissure treatment has been investigated, and BMI has not been observed to be an effective factor here [37]. However, another study evaluated pathologies that increase the frequency of complications after anal fissure surgery and found that age, female gender and a high BMI were associated with flatulence [38]. Another study also found that BMI was a risk factor for postoperative complications after anal fissure surgery. Obesity is more commonly associated with long-term complications [39].

Examining habits revealed that smoking prevalence differed between patients with acute and chronic anal fissures, with higher rates observed in the latter group. Notably, smoking rates are low in subgroups with inflammation and induration, which necessitate topical antibiotic therapy. This suggests that smoking may not be an aetiological cause. Poor hygiene and inadequate wound care are among the possible causes that come to the fore. However, a study evaluating the role of smoking in anal diseases observed it to be a risk factor in anal fissures and haemorrhoidal diseases [40]. However, in a study by Luc et al. addressing smoking and postoperative complications, smoking was not observed to be a risk factor [28]. Clearly, smoking needs to be examined in a single-variable study in terms of oxidative stress, impaired wound healing, oxygenation and perfusion disorders caused by vascular pathologies. This study examined the effects of age, BMI, lifestyle factors (e.g., smoking and alcohol consumption) and pain-related clinical variables on the group variable using multiple regression analysis. However, no variable was found to be a strong and independent predictor. The marginal relationships observed for BMI and smoking suggest that these factors should be re-evaluated using larger sample sizes or subgroup analyses.

Fissures should be treated before they become chronic. It is important to reduce pain and provide comfort to the patient. Although our study found no correlation between the treatment used and improvement in VAS scores, it should be remembered that many variables influence pain perception, such as confounding factors, sample size and concurrent treatments. As chronic fissures are more difficult to treat than acute fissures and have lower treatment rates, it is important to provide effective treatment during the acute phase [41]. These studies highlight the importance of treatment during the acute phase and emphasise that early and effective treatment is crucial in minimising the risk of complications. An anal fissure is a type of wound in the anal region. Therefore, a treatment plan that considers wound care, alongside pain palliation and symptomatic relief, should be established. In terms of the principles of wound healing, facilitating factors in chronic wound healing and delayed healing include bacterial overgrowth, insufficient or excessive vascularisation, and intense chronic inflammation [42,43]. Anal fissures can be considered wounds in which the normal healing process cannot be completed properly. The mechanism of anal fissure formation is similar to the predisposing factors of chronic wounds; therefore, treatment can be quite effective. Studies have shown that treating chronic anal fissures with povidone–iodine has a positive effect on the condition [44]. Therefore, based on clinical experience with anal fissure patients, topical antibiotic therapy has been incorporated into the treatment plan, given that infection can delay wound healing [45]. A study by Calomino et al. demonstrated that using topical calcium channel blockers and topical antibiotics for an appropriate duration after proctological surgery may improve symptoms and accelerate healing [46]. The important thing here is to determine which patient group needs it.

In this study, the interpretation of subgroup findings relies primarily on effect sizes and confidence intervals rather than statistical significance alone. Although topical antibiotic therapy demonstrated a larger and more precise effect in acute fissures, the confidence intervals in chronic fissures were wider and crossed unity, reflecting reduced precision rather than an opposite treatment effect. This uncertainty is likely due to a combination of the smaller sample size and the biological heterogeneity inherent in chronic wound pathology. Emphasising confidence intervals enables these findings to be interpreted more nuancedly and avoids the potential misinterpretation associated with post hoc power calculations. Subgroup effect size analysis revealed that the benefit associated with topical antibiotic therapy was similar in both acute and chronic anal fissures. However, the magnitude of the effect was greater and more robust in terms of statistics in acute fissures, whereas in chronic fissures, the confidence interval crossed unity. This suggests that biological factors related to chronic wound remodelling, fibrosis and impaired vascularisation, as well as limited sample size, may reduce the observable effects of treatment in chronic fissures. Notably, the consistent direction of the effect supports the hypothesis that antimicrobial modulation could contribute to mucosal healing across fissure types, with the greatest benefit observed when applied early in the disease process.

Our study found that the group receiving topical antibiotic therapy showed a high rate of mucosal healing, yielding results consistent with those of clinical studies. However, the lack of difference in subsequent treatment needs suggests that it would not be beneficial in the long term. As previous treatment modalities differed between groups in our study, the need for antibiotic therapy may have been due to the inadequacy of previous treatments. However, the fact that our patient group attended regular follow-ups reduces this possibility, further strengthening the view that it may be related to the shape and nature of the wound. Similar rates of improvement in VAS scores in the patient group receiving antibiotic therapy in addition to standard treatment, alongside a lack of differences between groups, indicate that, while antibiotic therapy promotes wound healing and is effective in treating bacterial infections, it does not provide patients with any analgesic benefit. It has been observed that pelvic floor exercises alone can lead to a significant improvement in VAS scores. Significant improvement is also inevitable with maximum medical treatment [47]. Another study evaluating VAS scores and topical analgesics discussed the overlap between pain treatment and concomitant disease treatment [48]. Several clinical studies have reported that the use of metronidazole in the treatment of acute anal fissure is associated with a significant reduction in pain severity and improvement in Visual Analog Scale (VAS) scores. Elgendy et al. investigated the effect of adding topical metronidazole to the standard treatment protocol for acute anal fissure and demonstrated accelerated epithelialization, more rapid pain relief, and consequently faster overall healing [49]. The pathophysiology of pain in acute anal fissure is multifactorial and involves internal anal sphincter spasm, local ischemia, inflammation, and individual variability in pain perception. Therefore, both the subjective pain experience and the response to treatment may vary considerably among patients. In clinical management, the therapeutic goal should extend beyond mere symptomatic relief and focus on correcting the underlying pathophysiological mechanisms and preventing recurrence through sustained therapeutic strategies. In this context, randomized controlled trials conducted in both acute and chronic anal fissure populations have shown that the addition of topical metronidazole to standard therapy results in faster relief of pain and sphincter spasm, as well as higher healing rates compared with control groups receiving standard treatment alone [50,51]. In light of current evidence, it may be concluded that adjunctive use of topical metronidazole alongside conventional therapies such as topical calcium channel blockers, nitrates, and warm sitz baths is associated with earlier reductions in pain scores and improved epithelialization in the short term. However, a critical consideration is the appropriate patient selection and indication for metronidazole therapy. Injudicious or excessive use may contribute to adverse outcomes, including the development of antimicrobial resistance.

Our study shows that the treatment of anal fissures involves multiple parameters and that no single treatment method is appropriate. A physical examination by an experienced practitioner is important. However, topical antibiotic therapy is not included in current guidelines for the treatment of anal fissures, despite its importance in complicated cases. It is also important to preserve the anal flora while ensuring wound healing. A study by Grekova et al. revealed that metronidazole, when used topically, was beneficial for patients with anal fissures when anaerobic bacteria were detected in stool samples [51]. In our study, antibiotic therapy was observed to be used in cases of inflammation, induration and minimal purulent discharge, which explains this situation. This helps to determine which cases are predicted to be or may be infected. It has been found to be more effective in acute anal fissures, while its effectiveness is more limited in chronic cases. This may be due to factors such as the deterioration of the flora or the inadequacy of topical treatment. Physicians’ physical examinations and direct observations also play a guiding role in selecting the appropriate treatment for the appropriate patient.

The limitations of our study are the lack of swab cultures from patients and the inability to make comparisons between groups. Therefore, we cannot discuss objective bacterial overgrowth. Another limitation is the lack of anoscopic examination or blinded assessment; complete mucosal healing was noted based on the clinician’s clinical opinion when following up with the patient.

## 5. Conclusions

In conclusion, the contribution of topical antibiotic treatment to mucosal healing in anal fissures, coupled with its sensitivity to previous treatment modalities, suggests that some patients may have received ineffective treatments in the past, that subclinical infection may be present, or that there may be differences in the factors affecting individual wound healing. In our study, we observed that some patients receiving standard treatment had mild signs of infection or wound care issues. Topical antibiotic treatment was considered beneficial for this group of patients. However, it was not effective in all patients with anal fissures; some cases required retreatment, and the distinction between acute and chronic fissures was observed to affect the treatment response. Our findings suggest that topical antibiotics are more effective when used appropriately during the acute phase. While antibiotic treatment may enhance mucosal healing, it is evident that this effect is not consistent across all patients, and definitive conclusions regarding microbial aetiology cannot be drawn without microbiological data. Therefore, unnecessary antibiotic use should be avoided, and treatment decisions should be based on careful patient selection.

## Figures and Tables

**Table 1:** Comparison of clinical and demographic characteristics of patients with acute and chronic anal fissure according to treatment groups.

Variables	Acute Fissure—Antibiotic (n = 22)	Acute Fissure—No Antibiotic (n = 36)	Chronic Fissure—Antibiotic (n = 27)	Chronic Fissure—No Antibiotic (n = 33)	*p*
**Age (years)**	42.63 ± 9.38	39.25 ± 13.47	42.18 ± 10.12	44.27 ± 9.29	0.616
**Male (n, %)**	11 (50%)	24 (66.7%)	18 (66.7%)	16 (48.5%)	0.665
**Female (n, %)**	11 (50%)	12 (33.3%)	9 (33.3%)	17 (51.5%)	
**BMI (kg/m^2^)**	25.20 ± 3.97	26.08 ± 5.19	25.57 ± 5.31	27.79 ± 4.96	0.092
**Number of smokers (n, %)**	5 (22.7%)	12 (33.3%)	8 (29.6%)	18 (54.5%)	**0.021** *
**Patients with complete mucosal healing (n, %)**	15 (68.2%)	13 (36.1%)	18 (66.7%)	14 (42.4%)	**0.024** *
**Patients requiring additional treatment (n, %)**	3 (13.6%)	7 (19.4%)	4 (14.8%)	9 (27.3%)	0.546
**Patients with prior treatment history (n, %)**	14 (63.6%)	9 (25.0%)	19 (70.4%)	10 (30.3%)	0.001 **
**VAS score improvement**	5.31 ± 1.70	5.25 ± 1.91	5.40 ± 1.59	4.48 ± 1.87	0.154
**Follow-up interval (months, mean)**	4.54 ± 2.55	5.19 ± 2.63	5.03 ± 2.54	5.09 ± 2.30	0.904

Singificance: * *p* < 0.05; ** *p* < 0.01

## Data Availability

The data that support the findings of this study are available from the corresponding author upon reasonable request.
